# Comparison of Gastric Cancer Risk Classifications Using Conventional and New Pepsinogen Criteria

**DOI:** 10.1155/2023/7646536

**Published:** 2023-05-30

**Authors:** Tae Sasakabe, Yuki Obata, Sayo Kawai, Yingsong Lin, Shogo Kikuchi

**Affiliations:** ^1^Department of Public Health, Aichi Medical University School of Medicine, Nagakute, Aichi, Japan; ^2^College of Pharmacy, Kinjo Gakuin University, Nagoya, Aichi, Japan

## Abstract

**Background:**

New serum pepsinogen (PG) criteria have been shown to indicate more accurately infection with *Helicobacter pylori* (*H. pylori*). We sought to improve risk classification for gastric cancer by adopting the new PG criteria with the addition of an *H. pylori* antibody test.

**Methods:**

The study participants were 275 patients with gastric cancer and 275 apparently healthy controls from case–control study data. We cross-sectionally compared the results of gastric cancer risk classifications that were based on a combination of the new PG criteria (PG II ≥ 10 ng/mL or PG I/II ≤ 5) and an *H. pylori* antibody test with those that were based on a combination of the conventional criteria (PG I ≤ 70 ng/mL and PG I/PG II ≤ 3) and an *H. pylori* antibody test.

**Results:**

Applying the conventional criteria resulted in 89 controls being classified as low risk. Applying the new criteria resulted in 23 controls (bootstrapped 95% confidence intervals [CI]: 14, 32) being additionally classified as high risk. Eight patients with gastric cancer were classified as low risk using the conventional criteria; however, six of these patients were classified as high risk by the new criteria (bootstrapped 95% CI: 2, 11).

**Conclusions:**

Compared with the conventional criteria, the new PG criteria with *H. pylori* antibody reduced instances of gastric cancer cases being misclassified as low risk. These findings suggest that the new PG criteria may help identify individuals at high risk of developing gastric cancer.

## 1. Introduction

At present, gastric cancer is the third most common cause of cancer death in Japan, accounting for about 42,000 deaths in 2020 [1]. Therefore, more efforts to reduce the high burden of gastric cancer are needed. National screening guidelines recommend gastric radiography or endoscopy for individuals over 50 years of age; however, participation rates for both types of examinations remain low [2, 3]. In particular, disparities in the medical resources needed for endoscopy limit its widespread use in population surveillance [4].

Given the resource-intensive nature of endoscopy, serology-based risk assessment with endoscopic follow-up has been proposed to aid in risk classification and early detection [5]. One current risk classification method (known as the “ABC” method) uses a combination of serum pepsinogen (PG) and *Helicobacter pylori* (*H. pylori*) antibody measurements [6–8]. Gastric cancer risk is classified into four groups, with group A (low risk) composed of individuals who test negative for both measurements. The remaining groups include individuals who test positive on both measures or either one of them. PG values reflect the severity of gastric mucosal atrophy and the degree of inflammation, both of which are associated with gastric cancer risk [9, 10]. PG I is primarily produced by the fundus and corpus areas of the stomach, whereas PG II is produced by all areas of gastric mucosa. When inflammation is present in the stomach, both PG I and PG II are elevated, with PG II showing a larger increase. When gastric atrophy occurs mainly due to continuous *H. pylori* infection, both PG I and PG II are reduced, with PG I showing a greater reduction. Based on the conventional PG criteria, an individual is considered to have gastric mucosal atrophy and high risk of gastric cancer if their PG I level is ≤70 ng/mL and their PG I/PG II ratio is ≤3 [8, 11]. Evidence demonstrating that gastric cancer is detected more frequently than expected in individuals classified as low risk is accumulating [12].

**Figure 1:**
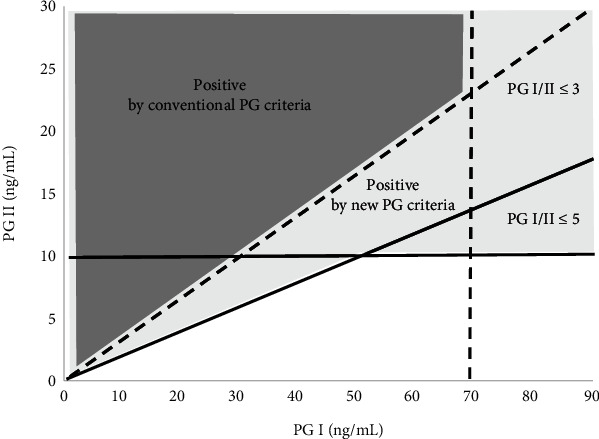
Scheme showing the relation between the new and conventional serum PG criteria. Dotted lines are cutoff values for the conventional PG criteria (PG I ≤ 70 ng/mL and PG I/PG II ≤ 3). Straight lines are the cutoff values for the new PG criteria (PG II ≥ 10 ng/mL or PG I/PG II ≤ 5). The light gray area shows the high-risk subjects based on the new PG criteria, whereas the dark gray area shows the high-risk subjects based on the conventional PG criteria. The new PG criteria are a necessary condition for the conventional PG criteria.

Meanwhile, it has been shown that PG values can also serve as a marker of *H. pylori* infection [13, 14]. Recently, optimal (new) criteria for PG values (PG II ≥ 10 ng/mL or PG I/PG II ≤ 5), which are based on the chemiluminescent magnetic particle immunoassay (CLIA) method, has been proposed to diagnose *H. pylori* infection [15]. Theoretically, the new criteria classify all individuals classified as high risk using the conventional criteria and some additional individuals as high risk (Figure 1). Therefore, using the new PG criteria in combination with *H. pylori* antibody could help prevent the overlooking of truly high-risk subjects, but increase the misclassification of truly low-risk subjects.

In 2003, we measured PG values and *H. pylori* antibody titers in our previous case–control study involving 275 patients with gastric cancer and 275 apparently healthy control subjects. On the basis of those results, we compared the results of gastric cancer risk classifications using the new PG criteria (with *H. pylori* antibody) with those using the conventional criteria (with *H. pylori* antibody). The aim of this study is to evaluate the merit (increase of accurate classification of gastric cancer patients) and demerit (increase of misclassified control subjects) when the new PG criteria are applied to the gastric cancer risk classification.

## 2. Methods

### 2.1. Participants

This is a cross-sectional study using data from a case–control study. The recruitment procedure has been described in a previous report [16]. Briefly, cases were newly diagnosed as gastric cancer in one of nine hospitals in the Tokyo area between 1993 and 1995. Patients who had undergone treatment for gastric cancer were excluded. Endoscopy was performed, and the diagnosis was confirmed by an examination of resection or biopsy specimens. Based on the criteria proposed by the Japan Gastric Cancer Society, gastric cancer cases were divided into two histological types: intestinal and diffuse. Apparently, healthy controls were recruited from participants who underwent medical checkups during the same recruitment period. Written informed consent was obtained from all participants, who were then asked to provide sera. We enrolled 788 patients with gastric cancer and 1007 controls. From this group, we randomly selected 275 cases and 275 controls considering sex and age. Sex and age (±2 years) were matched between cases and controls. The serum samples were frozen at −80°C until analysis. The reanalysis of the data for this study was approved by the Ethics Committee of the Aichi Medical University School of Medicine (No. 2019-158).

### 2.2. Measurement Methods

Radioimmunoassay (RIA; RIA BEADS KIT; Abbott Co. Ltd., Tokyo, Japan) was used to measure serum PG values. The measured value using the CLIA method has been shown to be correlated with that using RIA method, and these can be substituted for one another [17]. Serum *H. pylori* immunoglobulin G antibodies were measured by enzyme-linked immunosorbent assay (ELISA) using a commercial kit (J-HM-CAP; Kyowa Medex, Tokyo, Japan). The assay was performed according to the manufacturer's instructions, and the cutoff of 2.3 ELISA value (EV) recommended by the manufacturer for current *H. pylori* infection was used [18]. Titers ≥2.3 EV were defined as positive.

### 2.3. Gastric Cancer Risk Classification by Serum Tests

The conventional and new PG criteria and the cutoff value for *H. pylori* antibody were as stated above. The gastric cancer risk of each participant was evaluated using the serum antibody titer and the conventional or new PG criteria. Negative serum antibody and serum test were defined as low risk; otherwise, the subject was defined as high risk requiring an endoscopic examination. The results of the classification were compared between the two definitions in cases with gastric cancer and controls.

### 2.4. Statistical Analyses

For variables that were not matched between cases and controls, comparisons of the two groups were tested by chi-squared test for categorical variables and the Mann–Whitney *U* test for continuous variables. When comparing gastric cancer risk classification by serum tests using the new PG cutoff values, 95% confidence intervals (CIs) were calculated from 10,000 re-samplings (by bootstrapping) and presented as bootstrapped 95% CIs. All statistical analyses were performed by the R version 4.2.2 [19, 20].

## 3. Results

The median serum *H. pylori* antibody, PG I, and PG II levels were higher (<0.05 for all) and the PG I/PG II level was lower (<0.05) in patients with gastric cancer than in controls (Table 1). A comparison of gastric cancer risk classification results between the conventional and new PG criteria in combination with *H. pylori* antibody is shown in Table 2. If serum *H. pylori* antibody titer was ≥2.3 EV, it was defined as antibody-positive. If the PG value satisfied the criteria, it was defined as PG-positive. When both antibody and PG were negative, it was defined as low risk. In all other cases, it was defined as high risk. In total, 89 controls (32.4%) were classified as low risk using the conventional PG criteria, among whom, 23 (bootstrapped 95% CI: 14, 32) were classified as high risk using the new PG criteria; 66 (24.0%) were classified as low risk by both criteria. On the other hand, 8 (2.9%) patients with gastric cancer (1 intestinal- and 7 diffuse-type) were classified as low risk using the conventional PG criteria, 6 (bootstrapped 95% CI: 2, 11) of whom (1 intestinal- and 5 diffuse-type) were classified as high risk using the new PG criteria, and 2 (0.7%) as low risk by both criteria.

## 4. Discussion

The ideal gastric cancer risk classification of the 550 subjects is as follows. All 275 patients with gastric cancer were classified as high risk, and among the 275 controls, those who had a history of *H. pylori* infection were classified as high risk and those who did not were classified as low risk. When the new PG criteria as opposed to the conventional criteria were applied to patients with gastric cancer, the number of patients classified as high risk increased from 267 to 273 (97.1–99.3% of the 275 patients), whereas the number of controls classified as low risk decreased from 89 to 66 (32.4–24.0% of the 275 controls). Because the 23 controls (additionally classified as high risk) consisted of both subjects with a high and a low risk of gastric cancer, additionally misclassified controls by the new criteria were at most 23. One concern is that the increase in subjects who are classified as high risk and requiring confirmatory endoscopy may increase endoscopy-related costs and worsen manpower shortages. However, given the declining trends in the incidence of gastric cancer (3), the proportion of subjects classified as high risk would likely decrease, thereby justifying the costs of confirmatory endoscopy. One important aspect in regard to cost-effectiveness is that applying the new PG criteria could be expected to prevent the overlooking of truly high-risk patients.

The conventional PG criteria use PG I and the PG I/PG II ratio, which detects gastric mucosal atrophy, whereas the new PG criteria use PG II instead of PG I, which detects inflammation of the gastric mucosa due to *H. pylori* infection [15]. Therefore, the detection ability of PG values for gastric cancer may differ depending on the histological type. Among the patients with gastric cancer, 155 (about 56%) had the diffuse type. Of the eight patients classified as low risk by the conventional criteria, seven were the diffuse type. Compared with the intestinal type, diffuse-type gastric cancer develops from less atrophic gastric mucosa and is thought to be overlooked more frequently [21]. On the other hand, the new PG criteria reflect the presence or absence of *H. pylori* infection. Indeed, the application of the new PG criteria in combination with *H. pylori* antibody allowed the classification of six gastric cancer patients, including one with intestinal type cancer, into groups requiring further examination by endoscopy. Therefore, adopting the new PG criteria, which are indicative of *H. pylori* infection, may help identify both intestinal and diffuse cancers.

In addition to problems, such as regional disparities in medical resources for endoscopy, the declining trend in the prevalence of *H. pylori* and the consequent decrease in gastric cancer incidence increases the “number needed to treat” (the number of tests needed to identify a gastric cancer case) [4, 22]. To solve these problems and maintain or improve the efficiency of endoscopic examinations for gastric cancer, it may be necessary to prioritize examinations for patients with a high gastric cancer risk. Serological tests are safe and low-cost. In addition, serologic tests for gastric cancer risk classification may be effective in various clinical settings, such as in countries with limited medical resources and for individuals who are reluctant to undergo endoscopy [23]. Further studies using gastric cancer mortality as an endpoint are needed to evaluate the effects of adopting the new PG criteria on serology-based gastric cancer risk classification more accurately.

This study did have some limitations. First, the recruitment period was in the 1990s, so the prevalence of *H. pylori* infection, especially in controls, was thought to be higher than that in recent cases. Most of the gastric cancer cases had *H. pylori* infection, which is similar to the recent situation. As the prevalence of *H. pylori* infection has been declining [22], it is expected that the number of subjects classified as high risk for gastric cancer in the control group both under the conventional and new criteria will decrease. Second, as this work was conducted using data from a case–control study, it was impossible to calculate the positive and negative predicative values with the use of the new PG criteria. Furthermore, efforts are needed to validate the new criteria in other populations, given the observed variations in gastric cancer incidence rates, the prevalence of *H. pylori* infection, and other lifestyle factors, such as smoking and dietary habits [24].

## 5. Conclusions

In conclusion, gastric cancer risk classification using the new criteria with a combination of both PG values and *H. pylori* antibody reduced the overlooking of truly high-risk subjects compared with the conventional criteria. Furthermore, studies are needed to confirm the risk classification ability of the new criteria in the present population.

## Figures and Tables

**Table 1:** Characteristics of the cases and controls in the present study.

	Cases (*n* = 275)	Controls (*n* = 275)	*P-*value^§^
Sex			
Men (%)	142 (51.6)	142 (51.6)	
Women (%)	133 (48.4)	133 (48.4)	
Age (years)^†^	53.5 ± 10.5	53.6 ± 10.5	
Smoking			
Never	127 (46.2)	141 (51.3)	
Current or former	142 (51.6)	121 (44.0)	0.128
Unknown	6 (2.2)	13 (4.7)	
Alcohol intake			
Never	92 (33.5)	74 (26.9)	
Current or former	165 (60.0)	165 (60.0)	0.254
Unknown	18 (6.5)	36 (13.1)	
Serum *H. pylori* antibody (EV)^‡^	7.10 (4.63–10.39)	4.07 (1.56–6.54)	<0.001
PG I (ng/mL)^‡^	49.60 (31.55–68.90)	43.10 (32.55–55.60)	0.016
PG II (ng/mL)^‡^	19.10 (13.65–26.35)	12.50 (7.13–19.50)	<0.001
PG I/II (ng/mL)^‡^	2.54 (1.85–3.20)	3.73 (2.24–5.66)	<0.001

^†^Mean ± standard deviation.

^‡^Median value and interquartile range.

^§^Chi-squared test (not included unknown categories) for categorical variables and Mann–Whitney *U* test for continuous variables.

*H. pylori*, *Helicobacter pylori*; EV, enzyme-linked immunosorbent assay value; PG, Pepsinogen.

**Table 2:** Comparison of gastric cancer risk classification results between the conventional and new PG criteria in combination with *H. pylori* antibody.

Cutoff value for serum *H. pylori* antibody = 2.3 EV			Patients with GC					Controls				
			New PG criteria that are indicative of *H. pylori* infection					New PG criteria that are indicative of *H. pylori* infection				
			PG II ≥ 10 ng/mL or PG I/PG II ≤ 5					PG II ≥ 10 ng/mL or PG I/PG II ≤ 5				
			Low risk		High risk^†^			Low risk		High risk^†^		
			*n*	95% CI^‡^	*n*	95% CI^‡^		*n*	95% CI^‡^	*n*	95% CI^‡^	
							Total (GC risk classification)					Total (GC risk classification)
GC risk classification	Low risk		2	(0, 5)	6	(2, 11)	8	66	(53, 80)	23	(14, 32)	89
PG I ≤ 70 ng/mL and PG I/PG II ≤ 3	High risk^†^		0	(0, 0)	267	(261, 272)	267	0	(0, 0)	186	(171, 201)	186
		Total (new PG criteria)	2		273			66		209		

^†^If serum *H. pylori* antibody titer was ≥2.3 EV, it was defined as antibody-positive. If the PG values satisfied the criteria, it was defined as PG-positive. When both antibody and PG were negative, it was defined as low risk. In all other cases, it was defined as high risk.

^‡^Bootstrapped 95% confidence intervals from 10,000 re-samplings when the new PG cutoff values were applied.

PG, pepsinogen; *H. pylori*, *Helicobacter pylori*; EV, enzyme-linked immunosorbent assay value; CI, confidence interval; GC, gastric cancer.

## Data Availability

Data supporting this research article are available from the corresponding author on reasonable request.
